# CO adsorption on Pt(111) studied by periodic coupled cluster theory

**DOI:** 10.1039/d4fd00085d

**Published:** 2024-08-22

**Authors:** Johanna P. Carbone, Andreas Irmler, Alejandro Gallo, Tobias Schäfer, William Z. Van Benschoten, James J. Shepherd, Andreas Grüneis

**Affiliations:** a Institute for Theoretical Physics, TU Wien Wiedner Hauptstraße 8-10/136 1040 Vienna Austria johanna.carbone@tuwien.ac.at andreas.grueneis@tuwien.ac.at; b Department of Chemistry, Michigan State University East Lansing Michigan 48824 USA

## Abstract

We present an application of periodic coupled-cluster theory to the calculation of CO adsorption energies on the Pt(111) surface for different adsorption sites. The calculations employ a range of recently developed theoretical and computational methods. In particular, we use a recently introduced coupled-cluster ansatz, denoted as CCSD(cT), to compute correlation energies of the metallic Pt surface with and without adsorbed CO molecules. The convergence of Hartree–Fock adsorption energy contributions with respect to randomly shifted *k*-meshes is discussed. Recently introduced basis set incompleteness error corrections make it possible to achieve well-converged correlation energy contributions to the adsorption energies. We show that CCSD(cT) theory predicts the correct order of adsorption energies for the considered adsorption sites. Furthermore, we find that binding of the CO molecule to the top and fcc site is dominated by Hartree–Fock and correlation energy contributions, respectively.

## Abstract 2

Application of periodic coupled-cluster theory for CO adsorption energies on the Pt(111) surface. The adsorption energy at the top site is mainly electrostatic, while at the fcc site it is correlation-based. This difference might account for the challenges DFT faces with the CO puzzle.

## Graphical abstract

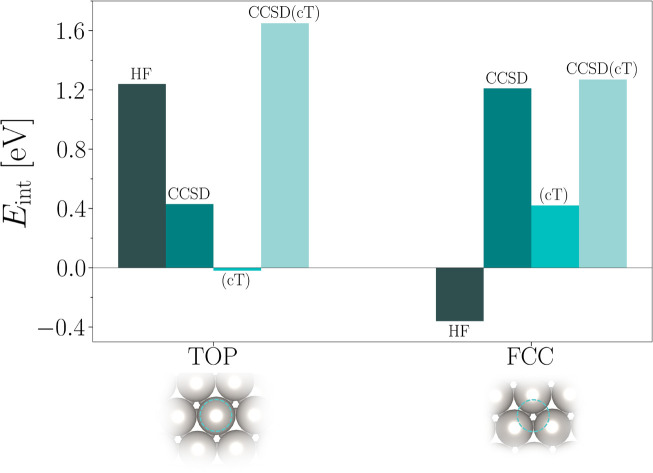

## 1. Introduction

The adsorption of a single CO molecule on the Pt(111) surface is a scientifically and technologically relevant chemisorption process, representing a prototypical reaction step in heterogeneous catalysis. Due to well-controlled low temperature measurements, both the adsorption energy and atomic structure can be determined with high precision.^1^ These and related experimental findings have been used intensely to benchmark a wide range of modern *ab initio* methods including approximate density functionals, many-electron perturbation theories and quantum Monte Carlo methods.

The seminal work of Feibelman *et al.* shows that the most widely-used local and semi-local density functionals predict the wrong adsorption site preference of CO on the Pt(111) surface compared to experimental findings at low temperatures.^2^ This puzzle and its far reaching consequences spurred the development of many (semi-empirical) corrections to the employed exchange and correlation density functionals. However, it was shown that achieving a simultaneous and accurate description of surface energies as well as chemisorption energies is impossible using state-of-the-art (semi-)local and hybrid density functionals.^3^ In contrast, a many-electron perturbation theory approach based on the random-phase approximation (RPA) solves this puzzle in a satisfactory manner.^3^ However, despite the recent advances of RPA calculations, there still exists the need for more accurate methods that go beyond the RPA. A well-established and highly accurate electronic structure theory is diffusion Monte Carlo, which has also been applied to the CO adsorption problem.^4,5^ Although both DMC studies predict the correct order of stability of CO adsorption sites, a relatively large discrepancy in the adsorption energy differences is observed. Alternative accurate *ab initio* methods would be helpful to resolve these and other relevant discrepancies.

In the field of molecular quantum chemistry, Coupled Cluster (CC) theories have established themselves as a class of highly accurate electronic structure theories that can achieve systematically improvable results depending on the level of truncation for the underlying particle–hole excitation operators. However, until recently their application to metallic systems was quite limited and mostly restricted to the level of Coupled Cluster Singles and Doubles (CCSD) particle–hole excitation operators.^6,7^ As shown in molecular quantum chemistry, highly accurate reaction energies require the inclusion of triple particle–hole excitation operators.^8^ The most popular triples approximation used in quantum chemistry accounts for these effects in a perturbative manner and is referred to as CCSD(T) theory.^9^ However, CCSD(T) yields diverging correlation energies for metals.^10^ Recently, a modified approximation to the triples, denoted as CCSD(cT) was presented.^11^ This method yields convergent and highly accurate results for the uniform electron gas.^11^

In this work we apply CCSD(cT) theory to the study of the CO adsorption on Pt(111) and test its reliability using a 2 × 2 surface slab model with 2 layers, which is sufficient to assess the qualitative level of accuracy for a range of electronic structure theories.

## 2. Computational methods

**Fig. 1:**
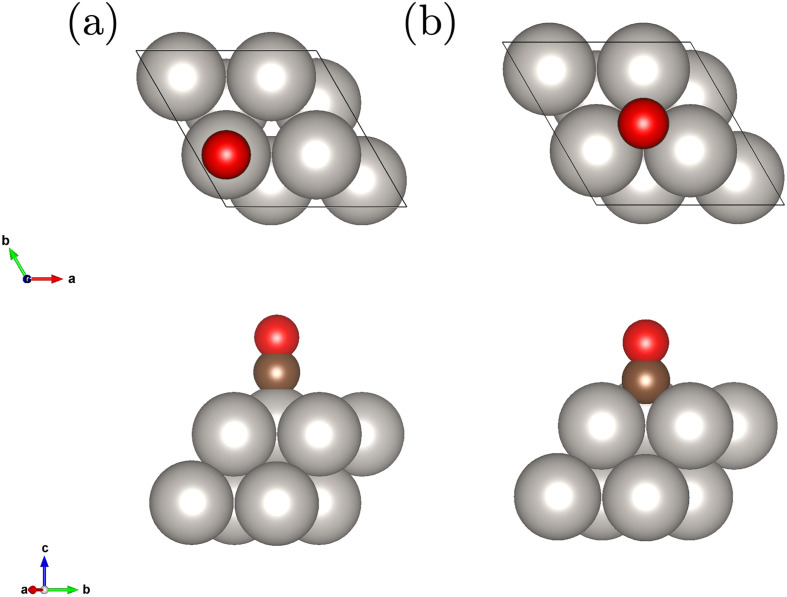
Employed Pt(111) surface slab model with 2 layers and the adsorbed CO molecule on the top (a) and fcc hollow site (b).

We study a 2 layer surface slab with 8 Pt atoms in the unit cell and an adsorbed CO molecule on the top and fcc hollow site depicted in Fig. 1. This corresponds to a coverage of 1/4. The geometries have been relaxed at the level of DFT-PBE.

The periodic coupled-cluster calculations reported in this work are performed using our high-performance open-source coupled cluster simulation code, Coupled Cluster For Solids (cc4s).^12^ The required reference wavefunction and the intermediates are obtained using the Vienna *ab initio* simulation package (VASP).^13–15^ For all calculations in VASP, a plane-wave kinetic energy cut off of *E*_cut_ = 600 eV is used. The Pt_GW, C_GW and O_GW POTCARS are employed. The smearing parameter is set to *σ* = 10^−4^ eV and a convergence criterion of Δ*E* = 10^−6^ eV is set for the self-consistent field methods. All Hartree–Fock (HF) calculations employ only integer occupation numbers and are performed using VASP.

In this work all post-HF calculations sample the first Brillouin zone using a single *k*-point only. Furthermore, the unoccupied HF orbitals for a given plane-wave basis have been computed by setting the number of orbitals/bands to the maximum number of plane-waves in the basis set. We note that the convergence of the CCSD correlation energy is very slow when canonical HF orbitals are employed. To accelerate the convergence to the complete basis set limit, we use approximate natural orbitals that are calculated using eqn (2) of the procedure outlined in ref. 16. After calculating all natural orbitals, a subset of them is chosen and recanonicalized. For coupled-cluster theory calculations we choose the number of unoccupied natural orbitals per occupied orbital to be in a range between 5 and 20. Additionally, for the basis set correction algorithm described below, the Moeller–Plesset Perturbation Theory (MP2) pair energies extrapolated to the complete basis set limit are needed, which are computed using the MP2 algorithm described in ref. 17. With this, VASP can provide all necessary files needed for the CCSD(cT) calculation, including the basis set error correction computed by cc4s. All calculations have been performed using about 8 compute nodes each equipped with 48 cores and 384 GB main memory.

All employed post-Hartree–Fock calculations use a finite number of particle states also referred to as virtual orbitals *N*_v_. The truncation of the virtual orbital basis set introduces a basis set incompleteness error (BSIE). The BSIE vanishes very slowly in the limit of *N*_v_ → ∞. In order to reduce the BSIE, we use a pair-specific cusp correction for CC theory.^18^ This scheme is based on frozen natural orbitals (FNOs) and diagrammatically decomposed contributions to the electronic correlation energy, which dominates the BSIE. To partly account for the BSIE of the (cT) contribution to the CCSD(cT) correlation energy, we rescale the (cT) contribution using the ratio of the MP2 correlation energy from the finite basis and the extrapolated complete basis set limit estimate. This correction was investigated on the level of (T) contributions and has been denoted (T*).^19^

Further, we simulate the Pt surface slab model using a periodic supercell approach with a finite size, introducing a finite size error (FSE). The HF and CC ground state energy converges slowly with respect to the system or *k*-mesh size. This partly follows from the fact that correlated wavefunction based theories capture longer ranged electronic correlation effects such as dispersion interaction explicitly. To reduce these FSE one can employ a correction method that takes advantage of the fact that the coupled cluster correlation energy can be expressed as an integral over the electronic transition structure factor multiplied by the Coulomb kernel in reciprocal space. Since finite size errors partly originate from an incomplete sampling of this integral in reciprocal space, an interpolation technique can be used to estimate and correct the FSE. The technical details are described in ref. 20. We note that this technique is not fully justified for metallic systems, which would require a linear interpolation of the transition structure factor in the long-range limit (seen in ref. 21 and 22). Therefore we have only applied this method for the calculated adsorption energies to investigate its order of magnitude. Our computations gave finite size corrections to the adsorption energies on the scale of 0.1 eV. Although this effect is small compared to the total adsorption energies and is not expected to change the reported results of this work in a significant manner, we note that future work will employ the RPA to account for this missing contribution in a more reliable way.

Throughout this work we discuss interaction energies that are defined as

1 −*E*^X^_Y_ = *E*^X^_CO@Y_ − *E*^X^_CO_ − *E*^X^_Pt(111)_,

where *E*^X^_CO@Y_, *E*^X^_CO_ and *E*^X^_Pt(111)_ correspond to total energies of the surface slab with the adsorbed CO molecule at site Y, the isolated CO molecule and the clean Pt(111) surface slab, respectively. Positive interaction energies indicate binding between surface and adsorbate. We restrict Y to the top and fcc hollow site depicted in Fig. 1. X indicates the employed level of theory. On the mean field level, we employ DFT-PBE (X = PBE) and HF (X = HF). All post-HF energies are computed from the sum of the HF energy and a correlation energy contribution. To facilitate a discussion of, for example, the convergence of correlation energy contributions with respect to basis set size, we use X = CCSD-corr. and X = (cT)-corr. when referring to the CCSD and (cT) correlation energy contribution, respectively.

Moreover, we discuss the difference in adsorption energies between top and fcc sites, which is defined as

2 −*E*^X^_top−fcc_ = *E*^X^_CO@top_ − *E*^X^_CO@fcc_.

Consequently, a positive value for *E*^X^_top−fcc_ indicates preference for the top adsorption site, which is experimentally found to be more stable. We note that all systems are computed using the same box size. Only for the CO molecule in the gas phase we employ a larger box until convergence is reached.

## 3. Results

We first discuss the obtained DFT-PBE results for the top and fcc interaction energies to assess the suitability of the employed surface model, consisting of a Pt(111) 2 × 2 surface slab with 2 layers only. Table 1 presents DFT-PBE interaction energies for 2 and 4 layers. We note that in both cases the fcc hollow site is the preferred adsorption site for the CO molecule with interaction energies of 1.94 eV and 1.73 eV. The fcc interaction energy for the 2 layer system is about 200 meV larger than for the 4 layer system. The same applies to the interaction energies for the top site with values of 1.82 eV and 1.61 eV. We emphasize that the difference between top and fcc interaction energies is 0.12 eV for both numbers of layers, indicating that this quantity converges fast with slab thickness. Although the surface model with two layers is smaller than those employed in, for example, ref. 3, our findings indicate that it already suffices to assess the ability of an electronic structure theory to predict the correct adsorption site preference. A comparison to PBE results from ref. 4 and 24 shows that our estimate of the DFT-PBE interaction energy differences agrees to within 10 meV even for larger coverages. For comparison we also show results from ref. 23 in Table 1, verifying our DFT-PBE estimates. An experimental estimate of the CO interaction energy with the surface at the top site with and without correction for vibrational zero-point energy contributions is reported in ref. 25 and amounts to 1.29 eV and 1.24 eV, respectively. Note that our definition of the interaction energy corresponds to the adsorption energy with an opposite sign. The range of experimentally measured interaction energies is relatively large, for example, ref. 4 compares to experimental estimates of about 1.43–1.71 eV.^1,26^ Without exploring the discrepancies in the reported experimental adsorption energies in greater detail, we conclude, in agreement with previous findings reported in the literature, that DFT-PBE predicts the wrong adsorption site preference and tends to overestimate interaction energies.

**Table 1:** Interaction energies of CO on Pt(111) for top and fcc hollow adsorption sites at the DFT-PBE level of theory using a 6 × 6 × 1 *k*-mesh. All energies in eV

Layers	*E* ^PBE^_top−fcc_	*E* ^PBE^_top_	*E* ^PBE^_fcc_
2	−0.12	1.82	1.94
4	−0.12	1.61	1.73
4		1.68 (ref. 23)	

Having established that the two layer Pt(111) surface slab model is sufficient to determine the order of stability of the considered CO adsorption sites, we now turn to the discussion of results obtained at the level of quantum chemical many-electron theories, starting with the HF approximation. It is important to note that the reported HF and post-HF calculations employ a reciprocal representation of the Coulomb kernel that was recently presented in ref. 27. This method samples the reciprocal Coulomb kernel consistently for arbitrary shapes of reciprocal volume elements, which is especially important for surface slabs. We stress that its treatment of the Coulomb singularity in reciprocal space is needed to achieve convergent adsorption energies in the present case. Moreover, we emphasize that convergent HF energies for metallic Pt(111) surfaces with and without adsorbed CO molecules can only be obtained when sampling the first Brillouin zone using *k*-meshes that are randomly shifted from high-symmetry points, *e.g.*, Γ. This behaviour is attributed to the presence of degenerate orbital energies at high-symmetry points that can not be properly accounted for in HF calculations using integer occupation numbers, leading to convergence problems of standard iterative self-consistent field solvers as implemented in VASP. However, as shown in ref. 6 and 11, we avoid these problems by averaging over HF energies computed using randomly shifted *k*-meshes. The standard error of the mean is employed as a measure of convergence for this Monte Carlo integration procedure over the first Brillouin zone.

Table 2 summarizes the obtained interaction energies and their difference at the level of HF theory for three different *k*-meshes. The first observation we report is that *E*^HF^_top−fcc_ converges faster than individual interaction energies with respect to the employed *k*-mesh size. Increasing the *k*-mesh from 3 × 3 × 1 to 4 × 4 × 1 changes *E*^HF^_top−fcc_ by 0.16 eV, which is in a similar order of magnitude as the error bars of the *E*^HF^_top−fcc_ 4× 4 × 1 result that originates from using about 10 random shifts in the calculations. It is noteworthy that HF theory predicts the experimentally observed order of stability for the top and fcc adsorption sites, with *E*^HF^_top−fcc_ ≈ 1.2 > 0 in contrast to *E*^PBE^_top−fcc_ = −0.12 < 0. *E*^HF^_top_ and *E*^HF^_fcc_ exhibit a slightly slower convergence with *k*-mesh size in the beginning. However, the changes from 3 × 3 × 1 to 4 × 4 × 1 are similar to *E*^HF^_top−fcc_. We note that the CO molecule is not binding to Pt(111) at the fcc site in HF theory.

**Table 2:** Interaction energies of CO on Pt(111) for top and fcc adsorption sites at the HF level of theory using three different *k*-meshes and twist averaging. The values in parenthesis give the standard error for the twist-averaging procedure. All energies in eV

*k*-mesh	*E* ^HF^_top−fcc_	*E* ^HF^_top_	*E* ^HF^_fcc_
1 × 1 × 1	1.29 (0.14)	0.69 (0.27)	−0.60 (0.28)
3 × 3 × 1	1.46 (0.11)	1.18 (0.08)	−0.27 (0.08)
4 × 4 × 1	1.62 (0.12)	1.24 (0.07)	−0.36 (0.16)

We now discuss the basis set convergence of the correlation energy contributions to the fcc hollow and the difference between top and fcc hollow interaction energies. Although we employ recently introduced corrections to reduce the BSIE of CCSD and (cT) correlation energy contributions, it is necessary to confirm that we employ basis set sizes that allow for achieving sufficiently well converged estimates. Tables 3 and 4 summarize CCSD and (cT) correlation energy contributions for different ratios *N*_v_/*N*_o_, respectively. Here, *N*_v_ represents the number of natural orbitals, and *N*_o_ denotes the number of occupied orbitals. It is shown that a value of *N*_v_/*N*_o_ = 10 is converged to within a few ten meV compared to slightly larger basis set sizes for *E*^CCSD-corr.^_fcc_ and *E*^(cT)-corr.^_fcc_. In the case of *E*^CCSD-corr.^_top−fcc_ and *E*^(cT)-corr.^_top−fcc_, this is already achieved even when compared to *N*_v_/*N*_o_ = 5, illustrating that the difference in adsorption energies benefits significantly from BSIE cancellation.

**Table 3:** Correlation energy contribution to the interaction energies of CO on Pt(111) for the fcc hollow adsorption site and the difference between top and fcc at the CCSD level of theory using a 1 × 1 × 1 *k*-mesh. All energies in eV

*N*_v_/*N*_o_	*E* ^CCSD-corr.^_top−fcc_	*E* ^CCSD-corr.^_fcc_
5	−0.74	1.62
10	−0.76	1.54
15		1.55

**Table 4:** Correlation energy contribution to the interaction energies of CO on Pt(111) for the fcc hollow adsorption site and the difference between top and fcc at the (cT) level of theory using a 1 × 1 × 1 *k*-mesh. All energies in eV

*N*_v_/*N*_o_	*E* ^(cT)-corr.^_top−fcc_	*E* ^(cT)-corr.^_fcc_
5	−0.44	0.40
10	−0.43	0.49
15		0.52

Having established that using *N*_v_/*N*_o_ = 10 suffices to achieve CCSD(cT) correlation energy estimates with BSIE smaller than a few ten meV, we choose to employ *N*_v_/*N*_o_ = 10 and seek to address the issue of convergence with respect to the number of randomly shifted 1 × 1 × 1 *k*-meshes. Table 5 summarizes the obtained CCSD and (cT) correlation energy contributions to the studied interaction energies and their difference. We employ 11 random shifts for the studied systems to obtain an average contribution. The values in the parenthesis correspond to the error of the mean, illustrating that the computed interaction energies show a relatively large dependence on the chosen shift. However, using a sufficiently large number of shifts allows us to achieve a statistically meaningful estimate of *E*^X^_top−fcc_, *E*^X^_top_ and *E*^X^_fcc_. It is noteworthy that the error of *E*^X^_top−fcc_ is smaller than the sum of the errors of *E*^X^_top_ and *E*^X^_fcc_ because we use the same random shift for different structures when computing differences. The larger errors for *E*^X^_top_ and *E*^X^_fcc_ indicate that these estimates depend more on the chosen random shift than *E*^X^_top−fcc_.

**Table 5:** Correlation energy contribution to the interaction energies of CO on Pt(111) for top and fcc adsorption sites at the CCSD, (cT) and CCSD(cT) level of theory using a 1 × 1 × 1 *k*-mesh. Ten randomly chosen *k*-mesh shifts are used. All energies in eV

X	*E* ^X^_top−fcc_	*E* ^X^_top_	*E* ^X^_fcc_
CCSD-corr.	−0.78 (0.07)	0.43 (0.25)	1.21 (0.18)
(cT)-corr.	−0.44 (0.11)	−0.02 (0.09)	0.42 (0.09)
CCSD(cT)-corr.	−1.21 (0.17)	0.41 (0.33)	1.62 (0.22)

The correlation energy contributions on the level of CCSD(cT) summarized in Table 5 are strongly negative for *E*_top−fcc_, indicating that correlation effects favor CO adsorption on the fcc site opposed to the HF theory preference for the top site.

**Fig. 2:**
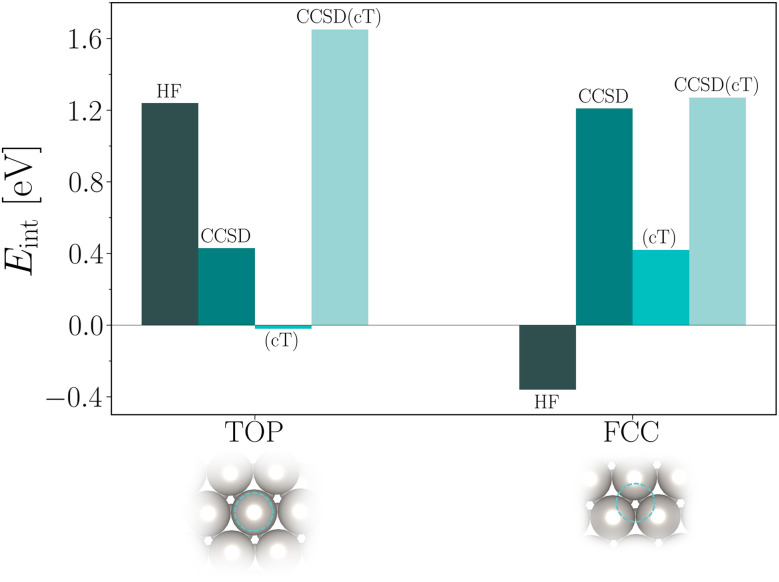
Bar plot showing energy contributions to the CO interaction energy on the top and fcc hollow site of Pt(111). HF disfavors and favors adsorption on the fcc and top site, respectively. CCSD and (cT) correlation energy contributions are also illustrated. The final estimates of the CO/Pt(111) interaction energies for the fcc and top site are given by the bars labelled CCSD(cT).

Finally, we discuss the obtained CCSD(cT) estimates for the CO interaction energies on Pt(111). Table 6 and Fig. 2 summarize all individual contributions already discussed above. We briefly reiterate that binding of CO to the top site is strongly favored on the level of HF theory. The additional CCSD(cT) correlation energy contribution to the interaction energy is about 0.5 eV. In contrast to the top site, HF theory predicts that CO is not bound at the fcc hollow site. Only when adding CCSD(cT) correlation energy contributions, the corresponding interaction energy becomes attractive with a significant contribution from the (cT) approximation. We note that the (cT) contribution for the CO adsorption to the fcc hollow site is relatively large indicating that intermediate to strong correlation effects could be involved.

**Table 6:** Final best estimates of contributions to the interaction energies of CO on Pt(111) for top and fcc adsorption sites at the HF, CCSD and (cT) level of theory. The complete CCSD(cT) estimate is also given. All energies in eV

X	*E* ^X^_top−fcc_	*E* ^X^_top_	*E* ^X^_fcc_
HF (4 × 4 × 1)	1.62 (0.12)	1.24 (0.07)	−0.36 (0.16)
CCSD-corr.	−0.78 (0.07)	0.43 (0.25)	1.21 (0.18)
(cT)-corr.	−0.44 (0.11)	−0.02 (0.09)	0.42 (0.09)
CCSD(cT)	0.41 (0.29)	1.65 (0.40)	1.26 (0.38)

Adding all contributions together gives *E*^CCSD(cT)^_top−fcc_ ≈ 0.4 eV, in qualitative agreement with recent DMC calculations yielding ≈0.25 eV (ref. 5) and ≈0.76 eV.^4^ RPA calculations yield a significantly smaller difference between adsorption energies of 0.08 eV.^3^ We note that the main uncertainty in our CCSD(cT) estimate originates from the random *k*-mesh shift averaging procedure. Future work will focus on reducing this error and will hopefully help to further resolve this discrepancy.

## 4. Conclusions

In conclusion we have computed interaction energies of CO with Pt(111) at the level of periodic coupled-cluster using a recently introduced set of methods, including: (i) corrections to the basis set incompleteness error,^18^ (ii) improved Brillouin zone sampling techniques ^6,27^ and (iii) the CCSD(cT) method, which accounts for triple particle–hole excitation operator correlation energy contributions using an approximation that averts the infrared divergence for metallic systems as observed for CCSD(T).^11^ This allows us to successfully converge the computed interaction energies with respect to the employed computational parameters, such as basis set size and *k*-mesh. The accuracy of CCSD(T) compared to experiment and high-level methods such as DMC has already been shown several times for CO adsorption energies on insulators such as MgO employing different approaches.^28–31^ The present work demonstrates an application of the related CCSD(cT) approach to the CO adsorption on a metallic surface.

The employed surface slab models using 2 layers are not large enough to allow for a direct comparison of all computed adsorption energies to experiment. Still using the 2 layer model, our CCSD(cT) interaction energy estimates for the top and fcc hollow site are 1.65 eV and 1.26 eV, respectively. Considering that, on the level of DFT-PBE we have estimated that the difference between 2 and 4 layers for the absolute adsorption energies are on the scale of about 200 meV, this would bring our estimates closer to experimental findings of about 1.29 eV that include zero-point vibrational effects.^25^ We also note that our findings for adsorption energy differences are already converged with respect to the number of layers and show a qualitative agreement with experiment and other high level theories.^3–5^ In particular, CCSD(cT) theory predicts the top adsorption site to be energetically more stable than the fcc hollow site by about 0.4 meV. This finding is larger than for the RPA results^3^ and lies between previously reported DMC findings.^4,5^ We emphasize that this brings periodic CC theories one step closer to play an important role in producing accurate benchmark results for technologically relevant surface chemistry problems studied in, *e.g.* heterogeneous catalysis.

An important observation of the present work is that the adsorption energy for the top site is dominated by electrostatic energy contributions, while the fcc site is dominated by correlation energy contributions. This in part might explain why most approximate exchange and correlation density functionals exhibit difficulties to predict the correct order of adsorption energies for different sites.^2^

Although the computational cost of the employed CC theories is significantly larger than that of standard DFT-PBE calculations, all required computations can be performed on a few modern multi-core compute nodes equipped with a few TB of main memory. Still, a remaining technical challenge that was identified in the present study is that standard iterative self-consistent field HF solvers converge frustratingly slow for metal surfaces, requiring up to several hundreds of steps until convergence is reached. In combination with the fact that we need to perform many HF calculations for different random *k*-mesh shifts, this creates a computational bottle neck that needs to be addressed in future work. Furthermore, the previously developed finite size correction method described in ref. 20, which highlighted the role of anisotropy, needs to be adapted to address the correct interpolation limit in a similar way to ref. 21 and 22 in order to account for the slower convergence of finite size error in metallic systems.^11^ However, we expect that the problems mentioned above can soon be resolved, paving the way for highly accurate CC theory calculations especially when combined with reduced scaling approaches such as suitable embedding techniques and local approximations.^31–34^

## Conflicts of interest

There are no conflicts to declare.
